# Tricuspid Valve Replacement With a Sewing Ring Extender to Address Conduction Abnormalities

**DOI:** 10.1016/j.atssr.2025.06.015

**Published:** 2025-07-21

**Authors:** Elizabeth L. Norton, Kanika Kalra, Robert A. Guyton

**Affiliations:** 1 Division of Cardiothoracic Surgery, Department of Surgery, Emory University School of Medicine, Atlanta, Georgia

## Abstract

Tricuspid valve surgery is associated with a high risk of new permanent pacemaker implantation. Tension on annular tissue by placement of a circular valve in a very noncircular annulus may cause conduction abnormalities. To address this, a triangular piece of pericardium was sewn to the prosthesis sewing ring, extending the sewing ring into the corner of the annulus between the septal and anterior leaflets, visibly relieving tension in the region of the atrioventricular node in five patients. A sewing ring extension of the prosthesis should be explored during tricuspid valve replacement to potentially reduce the risk of conduction system impairment.

Progressive tricuspid regurgitation (TR) is recognized as a serious threat to patient longevity and quality of life, leading to increasing interventions by both transcatheter and open surgical techniques.^1^^,^^2^ Open surgical tricuspid valve replacement (TVR) offers an opportunity to adapt the procedure to valve-specific pathology. But surgeons continue to place a round prothesis in a distinctly noncircular annulus. This leads to tissue distortion and visibly increased tension in the region of the conduction system. This region, the Achilles heel of the tricuspid annulus, is the cephalad portion of the triangle of Koch, adjacent to the cephalad half of the septal leaflet and the commissure between the septal and anterior leaflets. To avoid conduction tissue, valve sutures are frequently placed through leaflet tissue in this area,^3^^,^^4^ resulting too often in excessive tension causing tearing of the septal leaflet and subsequent deeper sutures hazardously close to the atrioventricular node. We suggest a flexible extension of the sewing ring to allow placement of a noncircular prosthesis matching the noncircular annulus to reduce tension on tissues near the atrioventricular node and potentially reduce the frequency of conduction abnormalities.

An 86-year-old female patient with a history of hypertension, atrial fibrillation, chronic left leg lymphedema, and progressive symptomatic right heart failure with severe tricuspid regurgitation presented to clinic for TVR evaluation. Transthoracic echocardiography revealed a very dilated right atrium, poor coaptation of the tricuspid leaflets, severe TR with a peak right ventricular pressure of 32 mm Hg, and a dilated tricuspid annulus at 5 cm. The size of her tricuspid annulus excluded her from all current approved and experimental transcatheter therapies and surgical TVR was recommended. She was admitted for elective minimally invasive TVR. An anterior right thoracotomy was created, and bypass was initiated via femoral arterial and venous cannulation. A wire-bound endotracheal tube was used for transatrial superior vena caval cannulation. The patient was cooled to 34°C, and the heart was not arrested. This technique eliminated any need for dissection of the cavae or of the aorta.

The modified bioprosthesis was prepared on the back table by suturing a triangular piece of bovine pericardium to the sewing cuff of a no 33 biologic valve (Figure 1A). The corner of a rectangular piece of off-the-shelf pericardium was sewn to the sewing cuff between 6 o’clock and 9 o’clock on the annulus from the surgeon’s view. This flap extended the sewing ring in this region by 8 to 16 mm, providing abundant length in the square corner of the new sewing ring to match the corner of the triangle of Koch between the septal and anterior leaflet. After right atriotomy and exposure of the tricuspid valve, horizontal mattress sutures were placed in the tricuspid annulus. In the area of the conduction system (corner of the annulus where the septal and anterior leaflet meet) sutures were placed through the septal leaflet and brought through the pericardial flap of the modified bioprosthesis (Figure 1B). The absence of tension on the sutures in the region of the anteroseptal commissure was easily visibly appreciated and there was no paravalvular leak on static testing. The postoperative course was uneventful: There were no conduction abnormalities, and the patient’s predischarge echocardiogram showed a well-seated valve and no paravalvular leak. She was discharged home on postoperative day 7.

**Figure 1:**
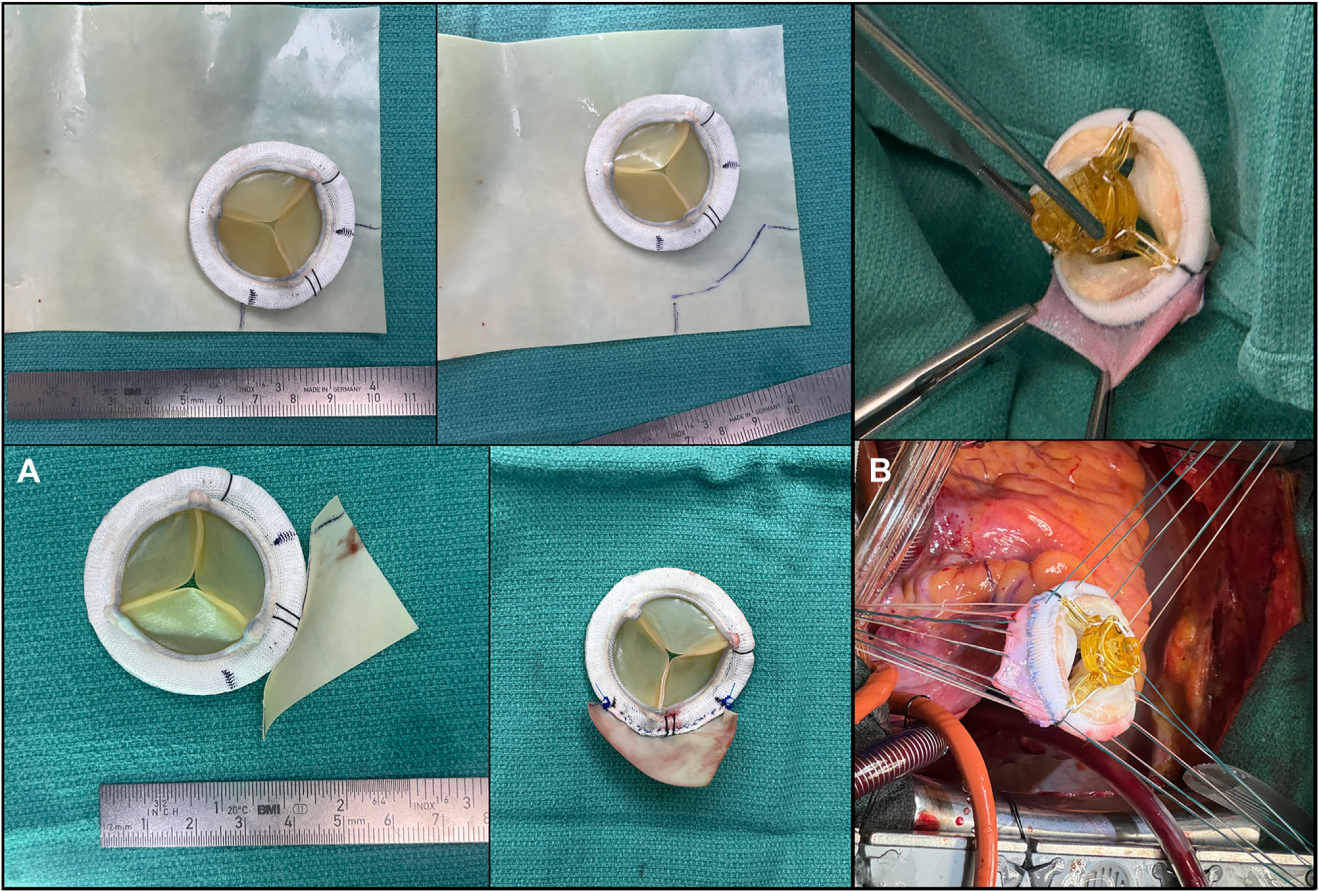
(A) Back-table preparation of the sewing ring extension on a bioprosthesis by suturing a triangular piece of bovine pericardium in running fashion to the sewing cuff of a biologic valve. (B) In the area of the conduction system, the corner of the annulus where the septal and anterior leaflet meet, valve sutures are placed through septal leaflet tissue and then through the pericardial flap (autologous pericardium in this case).

Since this initial case, 4 additional cases have been performed. All 5 cases were performed for severe TR: 2 for functional TR, 1 for primary TR, 1 for lead and pacemaker related TR, and 1 for endocarditis. In the endocarditis case, untreated autologous pericardium was used. The median age was 74 (range, 68-79) years, and 4 patients had atrial fibrillation. Median ejection fraction was 58% (range, 43%-60%). Surgical approach included sternotomy (2) and mini right thoracotomy (3) with a median cardiopulmonary bypass time of 126 (interquartile range, 99-133) minutes. Implanted valves included one 31-mm (Mitris, Edwards Lifesciences) and four 33-mm (Mitris [n = 2]; Epic Plus Mitral, Abbott [n = 2]). All patients had no or trace TR on chest closure transesophageal echocardiogram. One patient underwent planned pacemaker implantation (for preoperative tachy-brady syndrome), but no patients required pacemaker placement for conduction abnormalities postoperatively. Stroke, reoperation for bleeding, prolonged ventilation, and in-hospital mortality were 0%. All patients were discharged home with a median length of stay of 7 days. At time of follow-up echocardiogram (median, 4.4 months after surgery), 3 patients had no TR, 1 had trace TR, and 1 had mild TR with a median peak gradient of 3 (interquartile range, 2-4.6) mm Hg. Mortality was 0% at last follow-up (median, 7.3 months).

## Comment

Open surgical tricuspid valve surgery (repair and replacement) is associated with an embarrassingly high risk of requirement for a permanent pacemaker: 13% in repair and 25% in replacement in a recent Society of Thoracic Surgeons database study,^1^ which is associated with worse long-term survival.^2^ In an effort to decrease the risk of permanent pacemaker requirement in patients undergoing tricuspid valve replacement, we describe utilization of a sewing ring extender to reduce tension on sutures near the atrioventricular node. This technique can be utilized independently of approach (sternotomy and mini-thoracotomy) and with off-the-shelf supplies. When the surgeon chooses to place sutures through the septal leaflet to avoid the conduction system, the extension provides abundant tissue to relieve tension and potentially prevent tearing of the often-fragile leaflet. In the case of damage by endocarditis to tissue in the region of the atrioventricular node, this extension allows tissue to place sutures without excessive tension around the area of damage. Moreover, based on preoperative estimation of annular size and choice of prosthesis size, this extension can be created before establishing cardiopulmonary bypass to save time. This technical modification of the bioprosthesis is a simple yet effective strategy and might be considered in all patients undergoing tricuspid valve replacement.

In conclusion, modified bioprosthesis with a sewing ring extender of pericardium decreases tension on annular tissue near the conduction system during TVR and could reduce the incidence of required permanent pacemaker implantation.
